# Examining Ageism in U.S. Residency Programs: Nationwide Survey Using the Fraboni Scale of Ageism

**DOI:** 10.51894/001c.144197

**Published:** 2025-09-30

**Authors:** Megan Thomas

**Affiliations:** 1 College of Osteopathic Medicine Michigan State University

## 54

### INTRODUCTION

Ageism, defined as prejudice and discrimination against older adults based on age, is a significant concern in healthcare. Implicit biases among medical professionals can contribute to underdiagnosis, misdiagnosis, and delayed treatment, ultimately impacting the quality of care for older adults. As the aging population grows, it is essential to assess ageist attitudes in medical residency programs to ensure future physicians provide equitable and inclusive care. This study examines the prevalence of ageism among U.S. medical residents using the Fraboni Scale of Ageism.

### METHODS

A nationwide survey was conducted among U.S. residents in Internal Medicine, Family Medicine, and Psychiatry. The Fraboni Scale of Ageism, a validated 29-item questionnaire utilizing a four-point Likert scale, was used to assess ageist attitudes. Resident contact information was collected via FREIDA, and surveys were distributed through university email lists. Data was collected via Qualtrics and analyzed using Chi-square analysis and ANOVA.

### RESULTS

A total of 93 residents participated in the survey. The mean total Fraboni Scale score was 53 [range: 32-82], with subscale scores for anti-locution (9-31), discrimination (14-29), and avoidance (10-28). Findings indicated that residents trained under board-certified geriatric subspecialists exhibited lower ageist attitudes, were more comfortable with older adults maintaining their independence, and viewed them as more individualistic. Age was inversely related to ageism scores, with older residents demonstrating fewer ageist attitudes. Regional differences emerged, with Midwestern respondents demonstrating stronger discomfort with elderly cohabitation. Additionally, ethnicity influenced opinions on elderly suicide and friendship with older adults.

### CONCLUSION

Ageism in healthcare remains a notable concern, influencing patient care and medical decision-making. Training under geriatric specialists appears to mitigate ageist attitudes, emphasizing the importance of geriatrics education in residency programs. Future directions should include incorporating educational modules focused on reducing ageist biases and advocating for policy changes to improve geriatric training and care.

